# Transarterial radioembolization: a systematic review on gaining control over the parameters that influence microsphere distribution

**DOI:** 10.1080/10717544.2023.2226366

**Published:** 2023-06-21

**Authors:** T. J. Snoeijink, T. G. Vlogman, J. Roosen, E. Groot Jebbink, K. Jain, J.F.W. Nijsen

**Affiliations:** a Department of Medical Imaging, Radboud University Medical Centre, Radboud Institute for Health Sciences, Nijmegen, The Netherlands; b TechMed Centre, Multi-Modality Medical Imaging Group, University of Twente, Enschede, The Netherlands; c Faculty of Engineering Technology, Department of Thermal and Fluid Engineering, University of Twente, Enschede, The Netherlands

**Keywords:** Radioembolization, TARE, microspheres, distribution, computational fluid dynamics, CFD

## Abstract

*[Purpose]* Transarterial radioembolization (TARE) is an established treatment modality for patients with unresectable liver cancer. However, a better understanding of treatment parameters that influence microsphere distribution could further improve the therapy. This systematic review examines and summarizes the available evidence on intraprocedural parameters that influence the microsphere distribution during TARE as investigated by in vivo, ex vivo, in vitro and in silico studies. *[Methods]* A standardized search was performed in Medline, Embase and Web of Science to identify all published articles investigating microsphere distribution or dynamics during TARE. Studies presenting original research on parameters influencing the microsphere distribution during TARE were included. *[Results]* A total of 42 studies reporting a total of 11 different parameters were included for narrative analysis. The investigated studies suggest that flow distribution is not a perfect predictor of microsphere distribution. Increasing the injection velocity may help increase the similarity between flow and microsphere distributions. Furthermore, the microsphere distributions are very sensitive to the radial and axial catheter position. *[Conclusion]* The most promising parameters for future research which can be controlled in the clinic appear to be microsphere injection velocity as well as the axial catheter position. Up to now, many of the included studies do not take clinical feasibility into account, limiting the translation of results to clinical settings. Future research should therefore focus on the applicability of in vivo, in vitro, or in silico research to patient specific scenarios to improve the efficacy of radioembolization as treatment for liver cancer.

## 1. Introduction

With approximately 900,000 new cases and 830,000 deaths reported annually, primary liver cancer is the third leading cause of cancer death worldwide (Sung et al., 2021). In addition, each year one third (637,000 cases) of all patients suffering from colorectal cancer present with, or subsequently develop, colorectal liver metastases (Sung et al., 2021; Siebenhüner et al., 2020).

Curative treatment options, reserved for early-stage disease, include surgery (both tumor resection and liver transplantation) and locoregional therapies such as ablation. For advanced and metastatic disease, systemic therapy, transarterial chemoembolization (TACE) and transarterial radioembolization (TARE) can be considered (Su et al., 2022). Systemic treatment is the therapy of choice for widespread metastatic disease. However, it has a relatively high complication rate, and therefore there is an ongoing effort to develop more effective and less toxic locoregional treatment approaches for patients with oligometastatic disease, including TACE and TARE (Hilgard et al., 2010). These therapies are focused on preventing local progress and cannot systemically control tumor growth.

Nowadays TARE is increasingly considered for patients ineligible for curative therapy (Hilgard et al., 2010). TARE is based on the fact that both primary and secondary liver tumors are predominantly supplied by the hepatic artery, while healthy liver parenchyma receives most of its blood supply from the portal vein (Breedis and Young, 1954; Schenk et al., 1962). Therefore, radioactive microspheres are injected into the hepatic artery via a microcatheter and lodge predominantly in and around the tumor to deliver a high local radiation dose (Reinders et al., 2019).

Although TARE is an established treatment, uncertainty remains about the distribution of the microspheres throughout the liver (Caine et al., 2017; Salem et al., 2013). Smits et al. (Smits et al., 2013) found a tumor to non-tumor (T/N) ratio < 1.0 in 31 of the 107 tumors (29%). More recently, Garin et al. (Garin et al., 2021) pointed out that due to the absence of personalized dosimetry, the tumor absorbed radiation dose needed to achieve an effect is often not reached. However, treatment parameters concerning the delivery technique (e.g. number of administered microspheres, injection location, injection velocity), and the materials used (e.g. microsphere type, catheter type) (Caine et al., 2017; Kennedy et al., 2010) could be optimized to obtain higher T/N ratios. A growing body of research investigates such parameters using in vivo and in vitro experiments or in silico (computer) simulations. These works can be considered a first step toward personalized TARE treatment in which the parameters are chosen based on patient anatomy and physiology to optimize the dose distribution.

A recently published review by Aramburu et al. (Aramburu et al., 2022) gives an overview of Computational Fluid Dynamics (CFD) models used for TARE. These CFD models are based on numerical techniques to predict fluid flow and microsphere transport. In this review we complement and extend the previous work by focusing not on the models themselves but on the parameters that influence microsphere distribution and therefore the dose distribution during TARE, as investigated in vivo, in vitro and in silico. We emphasize the (dis)agreements within different types of experiments, discuss clinical relevance of the parameters and highlight open areas of research. By creating an overview of parameters which can be optimized for the individual patient, we aim to improve the dose distribution and patient outcome.

## 2. Methods

### Search strategy

This systematic review follows the guidelines of the Preferred Reporting Items for Systematic Reviews and Meta-Analysis (PRISMA). A search was conducted on the Medline, Embase and Web of Science databases for articles published online before September 29^th^, 2022. Articles whose abstracts contained synonyms of TARE in combination with synonyms for microsphere distribution or dynamics were included. The complete search strategy is accessible in Supplemental material 1. The article titles and abstracts were screened independently by two investigators (T.J.S. and T.G.V.). Studies meeting the inclusion criteria were selected for full-text screening. Any disagreements were resolved by consensus, and if consensus was not reached, a third investigator (E.G.J.) was consulted. Retrieved articles were imported into EndNote and duplicates were removed. The articles were then evaluated against a set of inclusion and exclusion criteria.

### Inclusion criteria

In vivo, ex vivo, in vitro and in silico studies that included original research on the microsphere distribution in TARE and were written in the English language were included for full text screening.

### Exclusion criteria

Articles were excluded if they investigated microspheres for other applications than TARE. Reviews, case reports, comments, editorials, and study protocols were also excluded. For the in silico studies, only models using full 3D computational fluid dynamics (CFD) simulations were considered.

### Study selection

After full-text screening, reference lists of all included articles were used for manual cross-referencing. All selected studies were subdivided into four groups: in vivo, ex vivo, in vitro and CFD studies.

### Analysis

No meta-analysis was performed on the selected articles due to the heterogeneity in study designs within the specific topics (in vivo, ex vivo, in vitro and CFD). Instead, a narrative analysis was performed.

## 3. Results

### The selected literature

**Figure 1.**
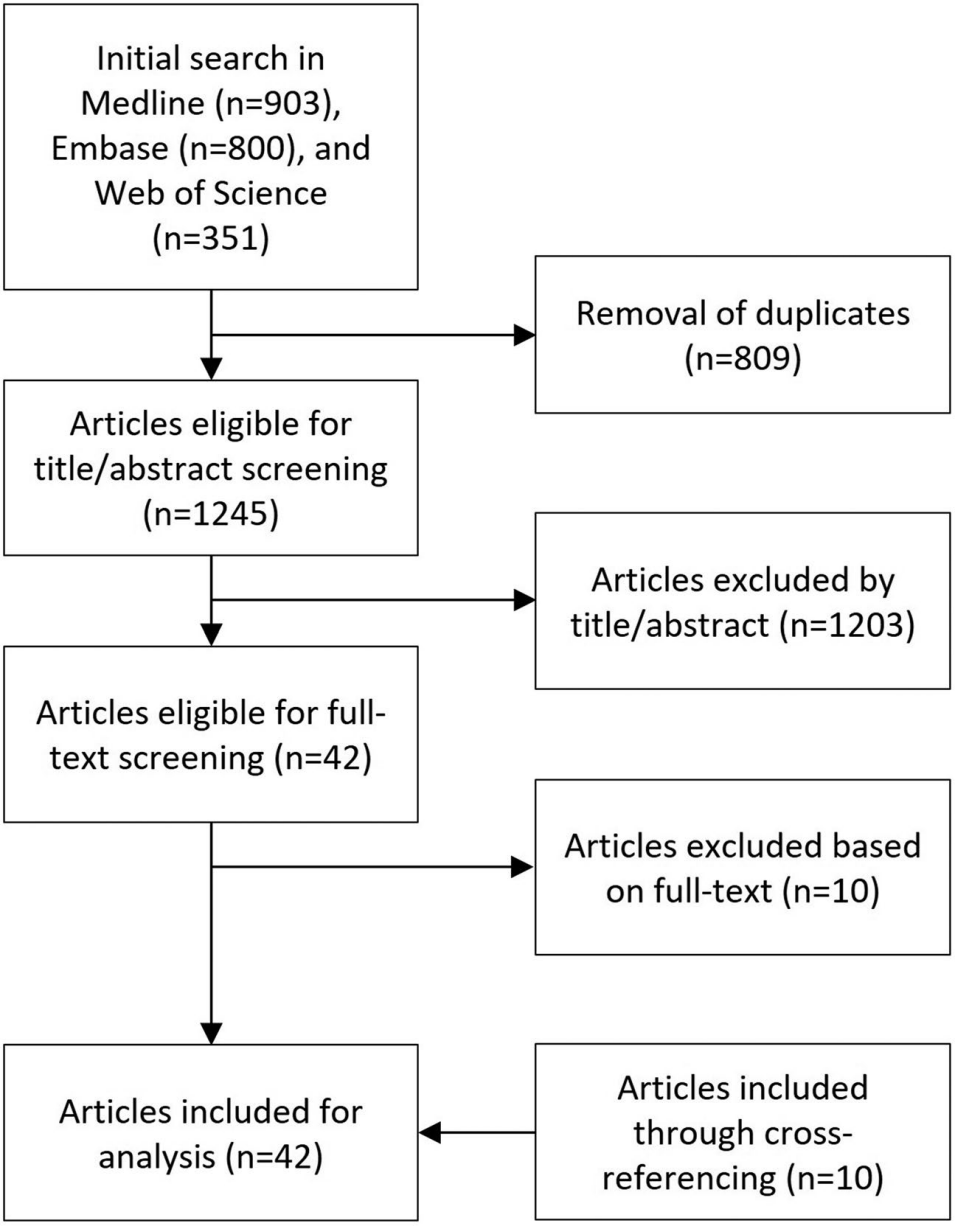
Flow chart of the selection process for articles investigating the microsphere distribution in TARE.

Our search strategy (Figure 1) resulted in 2054 articles, which was reduced to 1245 after removal of duplicates. In total, 42 publications were selected for final analysis (12 in vivo (Pasciak et al., 2015; van Roekel et al., 2021; D’Abadie et al., 2021; Meek et al., 2019; Rose et al., 2013; Burton and Gray, 1987; Burton et al., 1988; Burton et al., 1989; Burton et al., 1985; Rose et al., 2017; van den Hoven et al., 2016; Maxwell et al., 2022), 6 in vitro (Caine et al., 2017; Amili et al., 2019; Bomberna et al., 2021; Richards et al., 2012; Jernigan et al., 2015; Miller et al., 2022), and 24 CFD studies (Kennedy et al., 2010; Bomberna et al., 2021; Anton et al., 2021; Aramburu et al., 2017; Basciano et al., 2010; Childress et al., 2012; Lertxundi et al., 2021; Ortega et al., 2020; Roncali et al., 2020; Simoncini et al., 2017; Taebi et al., 2020; Taebi et al., 2021; Basciano et al., 2011; Kleinstreuer et al., 2012; Taebi et al., 2021; Aramburu et al., 2016; Aramburu et al., 2016; Aramburu et al., 2017; Childress and Kleinstreuer, 2014; Childress and Kleinstreuer, 2014; Aramburu et al., 2015)). Most frequently investigated parameters were microsphere type (7/37), injection related parameters such as injection velocity (8/37), catheter type (4/37) and location (radial 17/37, axial 5/37).

### 3.1. In vitro and CFD models

#### 3.1.1. General

Six in vitro studies investigated microsphere distribution in surrogate hepatic arterial systems. Furthermore, two studies used in vitro models as validation for their CFD model (Kennedy et al., 2010; Bomberna et al., 2021), from the total of 24 studies describing CFD models. Table 1 provides an overview of the investigated parameters and their influence on microsphere distribution. Additional data on all in vitro and CFD models are given in Supplemental material 2.

**Table 1.** Overview of studies investigating parameters and their influence on the microsphere distribution.

Article	Year	Study design	Radial injection location	Microsphere injection velocity/ technique	Microsphere type	Cancer burden / outflow distribution	Axial injection location	Catheter type	Model geometry	Injection timing in cardiac cycle	Systemic flow rate	Gravity	Viscosity injection solution
(Richards et al., 2012)	2012	In vitro	✓										
(Jernigan et al., 2015)	2015	In vitro		X	✓						X	X	
(Caine et al., 2017)	2017	In vitro		✓	X							X	✓
(van den Hoven et al., 2015)	2015	In vitro	✓					✓					
(Amili et al., 2019)	2019	In vitro				✓					✓		
(Miller et al., 2022)	2022	In vitro		✓									
(Kennedy et al., 2010)	2010	CFD	✓			✓							
(Basciano et al., 2010)	2010	CFD	✓		X					✓			
(Basciano et al., 2011)	2011	CFD	✓	✓	✓	✓				✓			
(Kleinstreuer et al., 2012)	2012	CFD	✓	✓					✓				
(Childress et al., 2012)	2012	CFD	✓						✓	✓			
(Childress and Kleinstreuer, 2014)	2014	CFD	✓							✓			
(Childress and Kleinstreuer, 2014)	2014	CFD	✓										
(Aramburu et al., 2016)	2016	CFD	✓			✓	✓	✓					
(Aramburu et al., 2016)	2016	CFD	✓	✓									
(Aramburu et al., 2017)	2017	CFD	✓		X	✓	✓						
(Aramburu et al., 2017)	2017	CFD		✓									
(Ortega et al., 2020)	2020	CFD	✓	✓	X								
(Taebi et al., 2020)	2020	CFD				✓							
(Bomberna et al., 2021)	2021	CFD	✓		X	✓	✓		✓				
(Ortega et al., 2022)	2021	CFD						✓					
(Taebi et al., 2021)	2021	CFD					✓						
(Taebi et al., 2022)	2022	CFD	✓				✓						
Ortega et al., 2022)	2022	CFD	✓					✓					
(Bomberna et al., 2022)	2022	CFD	✓						✓				
X times investigated			17	8	7	7	5	4	4	4	2	2	1

Notes: Empty cell: parameter not investigated in the study. ✓ Parameter impacts microsphere distribution. X Parameter was researched, but had negligible influence on microsphere distribution.

#### 3.1.2. Geometry

**Figure 2.**
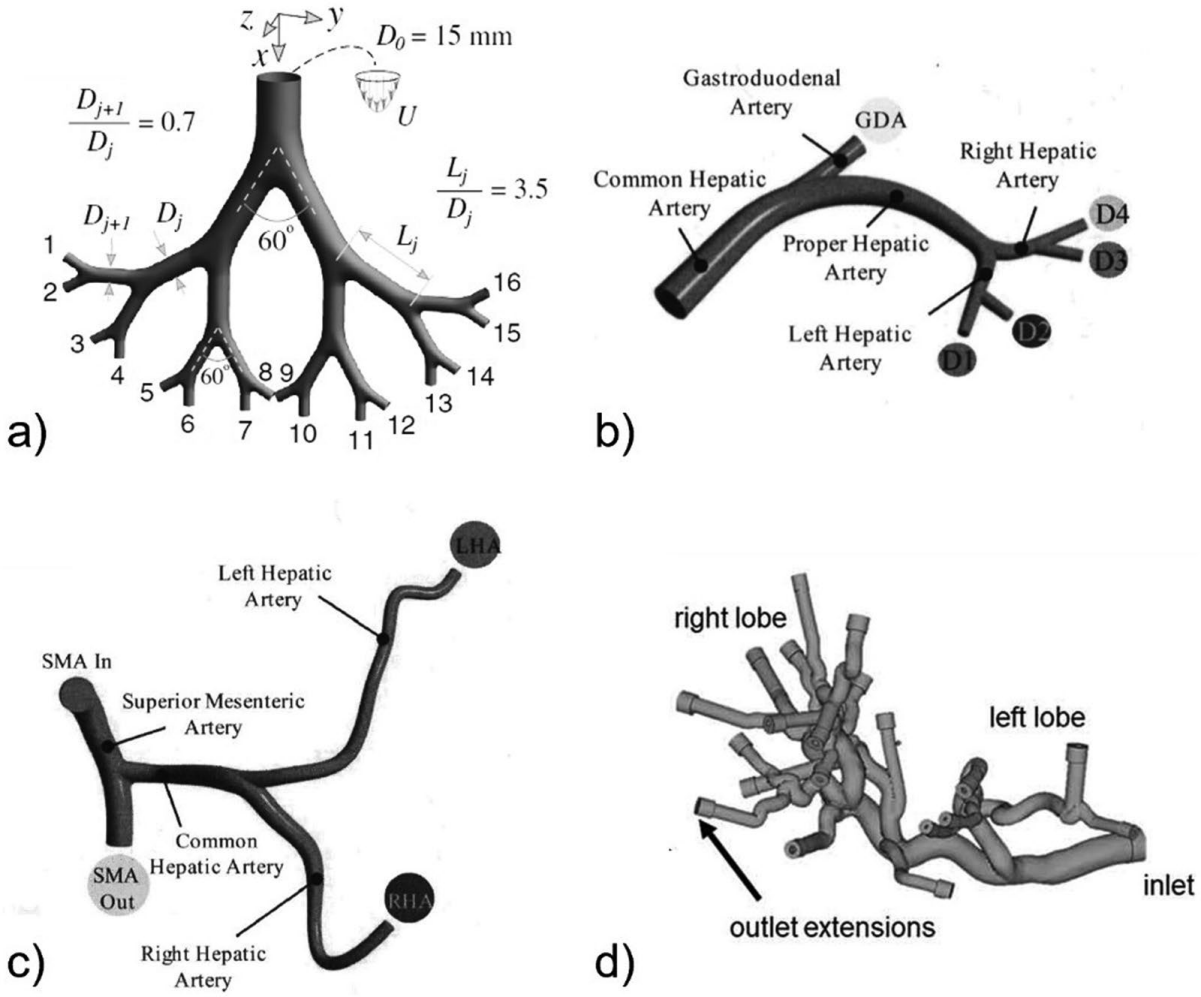
Examples of idealized and patient specific geometries. a) Idealized symmetrical geometry (Amili et al., 2019). b) basic arterial geometry (Childress et al., 2012). c) tortuous geometry (Childress et al., 2012). d) patient specific geometry (Bomberna et al., 2021).

The variation in TARE treatment outcomes is linked to the patient-specific anatomy of the liver vasculature. Comparison of different geometries can provide insight on the anatomical features that may influence microsphere distribution. Both patient specific and idealized liver models have been used to identify such features and their effects (Figure 2). To obtain idealized liver models, most studies use diameters known from published anatomic measurements of vessel diameters, sometimes in combination with 3D CT imaging (Jernigan et al., 2015) or angiographic images (Caine et al., 2017). The patient specific liver model used by Bomberna et al. (Bomberna et al., 2021) was obtained via vascular corrosion casting followed by micro-CT scanning. Anton et al. (Anton et al., 2021) used perfusion CT imaging to obtain patient-specific liver geometries, followed by planar angiography to determine the position of the catheter. For CFD simulations, the medical imaging data is usually segmented using commercial packages such as MeVis (Anton et al., 2021) or Mimics (Bomberna et al., 2021).

**Figure 3.**
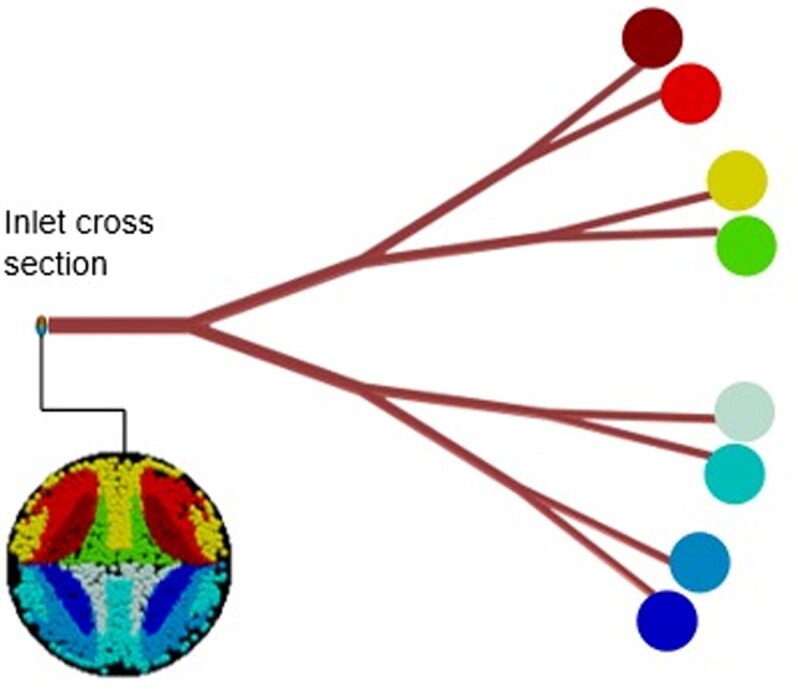
Example of a particle Release map (PRM). The cross section of the inlet plane is color coded; each color indicates the outlet that would be targeted if microspheres were injected at this particular radial location.

Four studies compared microsphere distributions in different geometries to gain insight into the features that make targeting a particular branch more easy or difficult. Kleinstreuer et al. (Kleinstreuer et al., 2012) and Childress et al. (Childress et al., 2012) compared microsphere transport using CFD models of the first few bifurcations. They advocated that tortuosity of the geometry impedes the targeting of particular branches. For the geometries shown in Figure 2(b,c), they constructed Particle Release Maps (PRMs): color-coded cross sections of the inlet plane which show the exit branches of the microspheres (Figure 3). The studies reported less clearly defined PRMs in the tortuous geometry (2c), making it more difficult to deliver particles to a particular branch by choosing an appropriate radial catheter position.

However, the work by Bomberna et al. (Bomberna et al., 2021) suggests that tortuosity alone cannot be said to determine the ease with which a branch may be targeted. This was quantified using the ‘targeting potential,’ defined as the degree to which a change in a particular injection parameter influences the distribution to a specific outlet. In their simulations of healthy (16 outlets) and cirrhotic (21 outlets) models, the *average* targeting potential over all outlets and all parameters was slightly higher for the (more tortuous) cirrhotic liver. However, the authors do not discuss which anatomical features influence the targeting potential at individual outlets.

Lertxundi et al. (2021) investigated three patient specific geometries with the goal of elucidating the role of simplifications in model geometry. By successively removing parts of the vasculature upstream of the catheter tip and downstream intra-segmental branches, they provided guidelines on which geometric simplifications do not affect the distributions. They were able to reduce computational time by an average of 62% while retaining distributions deviating less than 10% with respect to those obtained using the original geometry.

Bomberna et al. (2022) also investigated the effect of truncating the geometry to reduce computational effort. They found that modeling only the larger branches and assuming particles follow flow distributions in some of the smaller branches could lead to up to 27% less computational effort while outlet-specific differences in particle distributions deviated at most 3.5%.

#### 3.1.3. Flow characteristics

Arguably the biggest challenge in developing in vitro and CFD models which can be used to gain insight into the clinical situation is creating realistic modeling assumptions. In this section we discuss some of the assumptions that need to be made, what their effects on the microsphere distributions are and what must be considered to make an appropriate choice.

##### 3.1.3.1. In vitro

###### 3.1.3.1.1. Inlet characteristics

The impact of the chosen flow rate on the microsphere distributions was investigated in two in vitro studies (Caine et al., 2017; Amili et al., 2019) but remains largely unclear. Caine et al. (Caine et al., 2017) investigated a reduced flow rate (60 vs 120 ml/min) and found similar microsphere distributions across the range of flow rates for both glass and resin microspheres. However, Amili et al. (Amili et al., 2019) did find some differences in microsphere distribution when comparing flow rates corresponding to Reynolds numbers of 470 and 930 (larger Reynolds numbers correspond to larger flow rates). They found that at a higher flow rate flow separation occurred on the outer side of the bifurcations and expect that this hindered particles to follow the most extremal branches.

###### 3.1.3.1.2. Outlet characteristics

For in vitro models, the outlet diameter describes the degree of detail (macroscopic/microscopic level) with which the microsphere distribution is investigated. All models end in 5 to 21 outlets, with outlet diameters ranging from 0.9 − 1.7 mm. Amili et al. (Amili et al., 2019) used needle valves at each outlet to investigate the effect of doubling the flow rate in two of the outlets and found that an increase in flow rate to selected branches did not necessarily produce a proportional increase in particles, casting doubt on the contribution of tumor hypervascularity toward an increase in microsphere deposition.

##### 3.1.3.2. CFD

**Figure 4.**
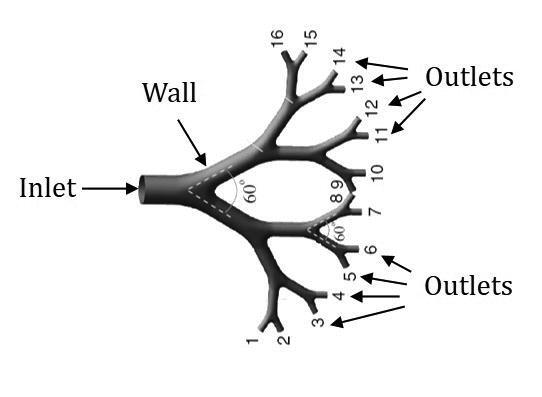
Inlet, outlet and wall boundaries. Boundary conditions need to be defined in a CFD model to perform physically realistic simulations (Amili et al., 2019).

Once a liver geometry has been obtained, a CFD model needs further specification of velocity and pressure related characteristics at the model boundaries. These *boundary conditions* at the inlets, outlets and arterial walls (Figure 4) are crucial to obtain physically realistic simulations.

###### 3.1.3.2.1. Inlet characteristics

The blood flow velocity profile in CFD models is often assumed to be uniform or parabolic throughout the cross-section. Ortega et al. (2020) investigated how the inlet velocity profile impacts the flow and particle distributions in an idealized (symmetric) hepatic geometry. They compared a flat inlet velocity profile with two ‘spiral-inflow’ profiles designed to mimic the helical flow that occurs in vivo due to the tortuosity of the arterial vessels. The helical flow profiles led to more rapid dispersion of particles across the cross section and very different particle distributions. This raises questions regarding the importance of an accurate inflow velocity profile when performing simulations in patient-specific geometries. However, this issue remains largely uninvestigated at present.

###### 3.1.3.2.2. Outlet characteristics

The boundary conditions at the outlets of a CFD model typically involve assuming outlet pressures, flow rates or resistances. The considerable influence of these boundary conditions on the flow is illustrated in the work by Kennedy et al. (Kennedy et al., 2010). Increasing the outlet pressure of the branch vessel in a simplified hepatic geometry (see Figure 2(b), where the branch vessel represents the gastroduodenal artery) by 20% reduced the flow rate to this outlet by 66%.

Two CFD studies (Bomberna et al., 2021; Aramburu et al., 2017) investigated the effect of increased flow rates at specific outlets, simulating the in vivo case of increased tumor tissue at those outlets. Bomberna et al. (Bomberna et al., 2021) increased the total tumor volume from 135 ml to 1129 ml, distributed over several outlets. Using a perfusion model, they derived the corresponding increase in flow rate to these outlets and found an increase in the number of particles exiting them. However, the size of this effect depends on the specific geometry of the patient as also noted in the in vitro work by Amili et al. (2019). In Bomberna’s work, the percentage of particles exiting the targeted branches under increased tumor load increased from 32% to 58% for tumors confined to the left lobe but only from 72% to 80% for tumors located in the right lobe.

Aramburu et al. (2017) used a similar approach with two cancer scenarios of 10% and 30% liver involvement to investigate to which extent the increased flow rate to cancerous outlets dominates the particle distribution. Increased cancer burden generally led to a decreased influence of other parameters such as injection location. For example, when injecting close to a bifurcation, a 5 mm axial shift in catheter position produced a maximum difference in per-outlet distribution of 39.74% in case of 10% liver involvement whereas this was only 12.60% for 30% liver involvement.

#### 3.1.4. Fluid for transport

##### 3.1.4.1. In vitro

It was investigated in vitro (Caine et al., 2017) whether a better match of the viscosity of the non-Newtonian Blood Mimicking Fluid (BMF) with the viscosity of the microsphere injection solution could result in enhanced mixing of the two fluids. Using different ratios of glycerol/water, different BMF viscosities were investigated (3.03, 4.00, and 9.89 cP). Increased viscosity of the injection solution, i.e. adding radiographic contrast agent to the saline injection solution, was found to require higher injection flow rates to achieve satisfactory mixing. Adding radiographic contrast agent in concentrations greater than 30% vol/vol produced diminishing levels of microsphere mixing with the BMF.

#### 3.1.5. Microsphere properties

##### 3.1.5.1. In vitro

Jernigan et al. (2015) found a significant difference in distribution between resin and glass microspheres while Caine et al. (2017) found the opposite. These contradicting outcomes may be explained by the scale at which the distributions were investigated. The smallest outlet branches in Caine’s model are of the order of 1 mm whereas Jernigan included a planar tumor model with diameters down to 20 µm. In vivo, most microspheres deposit in the arterioles (50–500 µm) and terminal arterioles (< 50 µm) (Hogberg et al., 2016). Jernigan et al. (2015) found a considerable influence of particle properties at a microscopic level by connecting a planar tumor model to one of the outlets and found that penetration depth of resin microspheres (29.1 µm, 1.57 g/mL) was significantly higher compared to glass microspheres (24.5 µm, 2.52 g/mL). In the scaled-up in vitro setup from Amili et al. (2019) particles of diameter 500 µm and 1000 µm were compared and it was found that larger particles favor branches with smaller branching angles due to their increased inertia.

Gravitational forces on particles might be expected to play a role (Gentile et al., 2008) as well in microsphere transport. Two studies (Caine et al., 2017; Jernigan et al., 2015) investigated the influence of gravity by rotating the geometry and found equivalent distributions for various angles, suggesting that gravity is of little influence.

##### 3.1.5.2. CFD

CFD simulations have shown a limited effect of microsphere properties on their distribution throughout the vasculature. Differences in trajectories between glass spheres with three times the density of their resin counterparts appear to be of consequence mostly in regions of extremely low flow velocity (Basciano et al., 2010; Ortega et al., 2020), where gravitational forces dominate the particle behavior. Ortega et al. (2020) suggests those velocities must be on the order of 1 0 ^−2 ^cm/s, which is lower than the velocities typically encountered in the hepatic vasculature at the height of the right and left hepatic artery (20–40 cm/s (Carlisle et al., 1992; Leen et al., 1991; Zoli et al., 1999)).

It has been suggested (Van de Wiele et al., 2012) that the size of particles also plays a role due to the Segre-Silberberg (‘skimming’) effect, in which smaller particles tend to travel more peripherally than larger ones. However, Aramburu et al. (2017) found that distributions of Technetium macroaggregated albumin (Tc-MAA) particles used in the pretreatment dose-estimation procedure (with half the diameter of the therapeutic glass or resin spheres) differed only between 1.64% and 5.87% for the various catheter positions and cancer scenarios investigated. The skimming effect therefore seems to play only a minor role.

#### 3.1.6. Injection method

##### 3.1.6.1. In vitro

The effect of injection velocity was tested in vitro by Jernigan et al. (2015). It was found that for glass microspheres the average penetration depth in the liver vasculature was higher for the maximum injection velocity (36.0 mL/min) compared to the minimum injection velocity (18.0 mL/min), though this result was not statistically significant.

More recently, the same group developed an in vitro model to investigate the effect of injection method (Miller et al., 2022). The effect on penetration depth in the tumor model was compared for two delivery devices: a dual-syringe (DS) system, which delivered a relatively constant microsphere concentration throughout administration and a bolus delivery (BD) system, which produced high microsphere concentration peaks early in the administration process (comparable to clinical practice). It was found that significantly less microspheres reached the distal microvasculature when using the BD system. The authors suggest that the BD system leads to increased back-pressure in the tumor, reducing tumor flow rate and leading particles to deposit in more proximal parts of the vasculature.

According to Caine et al. (2017), the degree of mixing of the injected microspheres and the blood will determine the homogeneity of the microsphere distribution in blood. Caine et al. evaluated the effect of microsphere injection velocity on mixing for constant injection velocities of 5-, 10-, 20-and 30-mL/min. At low injection velocities (5–10 mL/min) a laminar stream of injection solution was visible, which indicates inadequate mixing. Microsphere injection velocities > 10 mL/min enhanced mixing in BMF. To enable effective mixing, the flow rate at the catheter tip needs to be large enough to overcome the bulk flow inertia and viscous drag forces.

##### 3.1.6.2. CFD

The effect of injection velocity, location, and timing with respect to the cardiac pulse have been investigated using CFD models. Kleinstreuer et al. (2012) and Aramburu et al. (2017) both noted that relatively large injection velocities (approximately 5x larger) with respect to the bulk flow velocity led to particles crossing the flow streamlines. Aramburu et al. mentioned that larger injection velocities also led to faster mixing of the particles in agreement with the in vitro findings of Caine et al. (Caine et al., 2017). Increasing injection velocity thus appears to facilitate mixing of microspheres with flow but makes the sphere trajectories less predictable.

Multiple studies (Bomberna et al., 2021; Basciano et al., 2010; Childress et al., 2012) have shown the importance of axial injection location on the particle distribution in various geometries. Bomberna et al. (2021) reports that the particle distribution shows differences of a few percent between the different axial injection locations. However, this is under the assumption that the particles are uniformly distributed across the blood vessel inlet. The PRMs from the different axial injection locations show large differences from each other. Therefore, if injection would be performed within a small part of the inlet cross-section, the fraction of particles exiting each outlet is likely to be affected. Aramburu et al. (2017) and Taebi et al. (2022) found that the microsphere distribution is especially sensitive to changes in axial location when injecting close to a bifurcation. At such locations, the blood flow exhibits complex flow features which may make it difficult to target a particular outlet branch.

Basciano et al. (2010) investigated whether the moment of injection within the cardiac pulse affects the particle distributions in the simplified planar geometry first discussed by Kennedy et al. (2010). They found that choosing to inject either just before, during or after the moment of peak systolic flow rate had a large influence on the particle distribution amongst the four (targeted) daughter vessels.

#### 3.1.7. Catheter

##### 3.1.7.1. In vitro

The influence of a catheter on the flow profile is an important and largely unexplored area for research. The hepatic artery model described by Jernigan et al. (Jernigan et al., 2015) was used in a later study to investigate the effect of an anti-reflux catheter on particle dynamics (van den Hoven et al., 2015). This catheter features an expandable tip which is fixated in the center of the vessel lumen, preventing retrograde flow of microspheres. The anti-reflux catheter led to more chaotic outflow compared to a standard microcatheter. In addition, a significantly more homogenous distribution of microspheres in downstream branches was observed, which seemed to be related to the fixed radial position of the anti-reflux catheter tip. For the regular catheter, alignment of the tip is difficult to control; when the catheter tip was situated in the center of the vessel lumen, a homogenous distribution was found, while for the remaining experiments in which the catheter tip was off center, the distribution was skewed.

##### 3.1.7.2. CFD

Only some of the CFD studies (Aramburu et al., 2017; Childress et al., 2012; Kleinstreuer et al., 2012; Taebi et al., 2021; Aramburu et al., 2016; Aramburu et al., 2016; Aramburu et al., 2017) explicitly model the presence of a catheter, while all others release the particles downstream of the (hypothetical) actual injection location. Kleinstreuer et al. (2012) investigated the effect of a proposed ‘smart microcatheter’ (SMC) on the flow and particle distributions compared to the case where the catheter is not modeled. This SMC is kept in the desired radial position by a set of support struts. It was found that the catheter and support struts led to a local disturbance of the flow. Although the flow was only disturbed locally and damps out further downstream, this local disturbance did lead to an important alteration of the PRMs.

Aramburu et al. investigated the effect of two special types of microcatheters: the angled tip (Aramburu et al., 2017) and anti-reflux (Aramburu et al., 2016) microcatheter. Both catheter types aim to target a particular branch of the liver vasculature more effectively. In case of the anti-reflux catheter, it was found that this led to an important change in particle distributions when injecting far from a bifurcation but not so much when injecting close to it. For the angled tip microcatheter the effect of tip orientation was investigated. Intuitively one might expect that pointing the catheter tip toward the targeted branch will increase microsphere distribution to that branch by directing the particles initial momentum in that direction. However, the effect of the bulk flow was found to dominate that of the initial particle inertia and tip orientation was found to have only a small effect on the distributions.

Ortega et al. (2022) studied a novel type of catheter in which particles exit through the sides of the catheter. They found that such a catheter helps particles disperse over the vessel lumen more rapidly. This led to a more uniform concentration of particles in the flow which in turn increases the similarity between blood flow and microsphere distributions.

### 3.2. In vivo experiments

In vivo experiments are scarce as TARE is a highly patient-specific procedure and it is difficult to alter parameters within one patient. Nevertheless, the effect of catheter type is one of the parameters investigated in vivo (Pasciak et al., 2015; van Roekel et al., 2021; D’Abadie et al., 2021; Rose et al., 2013). The tip of an anti-reflux catheter expands during retrograde flow conditions, causing a decrease in blood pressure in the downstream hepatic compartments. One of the studies (Pasciak et al., 2015) mentions that the decrease in pressure causes vasoconstriction of the arteries and arterioles supplying normal liver tissue. Tumor arterioles, on the other hand, are not likely to vasoconstrict due to absence of adrenergic innervation (Ashraf et al., 1997; Mattsson et al., 1977).

The in vitro anti-reflux catheter experiments from Van den Hoven et al. (van den Hoven et al., 2015), were validated in a randomized controlled trial of Van Roekel et al. (van Roekel et al., 2021; van den Hoven et al., 2016). It was found that the anti-reflux catheter did not significantly increase the T/N ratio (median T/N ratio of 3.2 for the anti-reflux catheter vs median T/N ratio of 3.6 for the standard microcatheter). Moreover, technical adverse events frequently occurred, such as vasospasm in six of the 21 treated patients (29%) and catheter movement during deployment of the anti-reflux system.

Van Roekel et al. (van Roekel et al., 2021) contradicted the studies of d’Abadie et al. (D’Abadie et al., 2021) showing a significantly higher T/N ratio (median increase of 24%) for patients treated with an anti-reflux catheter (38 patients) compared to the standard microcatheter (23 patients). However, Van Roekel et al. used the difference in T/N ratio between the two catheters, with one type situated in the right lobe and the other in the left liver lobe, while d’Abadie et al. used the difference in T/N ratio between the preliminary scout dose injection and the anti-reflux guided therapeutic microsphere dose injection.

Balloon occlusion microcatheters have also been under investigation, concerning either proximal placement of a balloon microcatheter (Rose et al., 2017) to decrease blood pressure in the downstream compartments or distal placement with respect to the tumor-feeding vessels (Meek et al., 2019), to temporary redistribute flow. Both studies showed an increase in the number of microspheres toward targeted areas.

Another parameter investigated in vivo, was the number of particles in the treated volume (Maxwell et al., 2022). It was found that for hypovascular tumors a better Local Progression-Free Survival (LPFS) was achieved by delivering more particles (≥ 6000 particles/cm^3^ treatment volume), while for hypervascular tumors a better LPFS was achieved by delivering less particles (< 6000 particles/cm^3^). The authors hypothesize that for hypervascular tumors particles mostly flow to the tumor at the beginning. As the tumor gets saturated, particles start to flow to the healthy liver tissue indicating that the optimal endpoint is achieved. For hypovascular tumors on the other hand, more particles are needed to overcome poor tumor vascularity. However, the authors do not exclude that differences in the properties of the particles (glass vs. resin), the delivery method, or the differences in sensitivity for ischemia or radiation between hypervascular and hypovascular tumors may have an influence on the outcomes.

As a last parameter, the infusion of angiotensin II (AT-II) in the hepatic artery prior to the TARE procedure has been under investigation. Since tumor neovasculature lack immunoreactive nerves (Ashraf et al., 1997; Mattsson et al., 1977) the infusion of AT-II constricts normal liver arterial vessels and therefore reduces blood flow to normal liver tissue, whereas it is hypothesized to leave flow to tumor vessels relatively unaffected. Burton et al. found a significant increase in T/N ratio for AT-II and for the combination of noradrenaline and propranolol (Burton and Gray, 1987; Burton et al., 1988; Burton et al., 1985) in animals with implanted liver tumors. This was further investigated in a phase II clinical trial (Burton et al., 1989); however the exact influence of the AT-II remained unclear as there was no control group to which no vasoconstrictor was given. To the best of our knowledge, no other studies investigated the influence of AT-II infusion during TARE.

## 4. Discussion

### 4.1. Interpretation of the results

This review has summarized all original research into the influence of intraprocedural parameters on the microsphere distribution during TARE. The investigated studies mark important steps in increasing our understanding of radioembolization and improving treatment outcome by identifying the influence of parameters that can be controlled in the clinic. The complex nature of microsphere transport in a patient-specific hepatic geometry and a lack of quantitative measurements for many important model inputs (e.g. conditions at the inlet and outlets) make it a challenge to draw broadly applicable conclusions. However, the parameters injection velocity and radial and axial catheter position appear to most strongly affect the particle distributions based on the investigated studies.

The traditional view on TARE assumes that microsphere distribution largely corresponds to blood flow distribution. Because the percentage of arterial flow to tumorous tissue is higher than to normal tissue (Aramburu et al., 2016), this assumption implies that tumors receive a high local radiation dose while toxicity to healthy tissue is limited. However, the investigated studies suggest that a close correspondence between microsphere and flow distributions is not always achieved. The in vitro models by Amili et al. (2019) and Caine et al. (2017) as well as the CFD model by Kennedy et al. (2010) showed fundamental differences between microsphere and flow distributions. On the contrary, Bomberna et al. (Bomberna et al., 2021) found that microsphere distribution largely resembled flow distribution in their CFD model, though they also stated that significant differences were possible. Flow distribution is therefore related to, but not a perfect predictor for particle distribution as local flow structures (e.g. near the catheter tip) play a key role in determining the particle trajectories. Furthermore, the clinical parameters injection velocity and catheter position strongly influence the particle distributions.

Increased injection velocity was found to enhance mixing of microspheres and blood in the in vitro study from Caine et al. (2017), resulting in particle distributions more similar to flow distribution compared to low injection velocities. For clinical practice it is important to note that it seems that enhanced mixing can be nullified if high levels of radiographic contrast agent are used (> 30% vol/vol) (Caine et al., 2017). The enhanced mixing for increased injection velocities was confirmed in CFD simulations (Aramburu et al., 2017; Ortega et al., 2020; Basciano et al., 2011; Kleinstreuer et al., 2012; Aramburu et al., 2016). Due to its significant influence on the particle distributions, it could be valuable to more precisely control the injection velocity in clinical practice. For example, ensuring identical injection velocities between the preliminary scout dose injection and the therapeutic microsphere dose injection can be expected to make prediction of the radioactive dose distribution prior to treatment more accurate.

Although increased injection velocities help particles follow the flow distribution, it appears to make targeting *specific* branches more difficult. Three CFD studies (Kennedy et al., 2010; Basciano et al., 2010; Kleinstreuer et al., 2012), which all investigated the same geometry, concluded that the chaotic particle behavior stemming from large injection velocities prevents accurate branch targeting. The reason for this is that PRMs are mostly derived from simulations in which it is assumed that the blood flow is not disturbed by injection of particles. Large injection velocities create large flow disturbances near the catheter and therefore violate this assumption. Hence, to target a *specific* branch it may be more appropriate to inject with a velocity equal to the local flow velocity whereas a more general targeting strategy (in which particles should follow the flow distribution) might benefit from the increased mixing that occurs at larger injection velocities. This general targeting strategy is closer to clinical practice, in which it is the goal to distribute the microspheres equally to all branches in front of the catheter. To make specific branch targeting possible for the future, injection velocities matching the flow velocity should be considered.

The second important parameter is axial and radial catheter position, which had a large effect on particle distributions in the in vitro (Richards et al., 2012; van den Hoven et al., 2015) and CFD simulation (Kennedy et al., 2010; Bomberna et al., 2021; Basciano et al., 2010; Childress et al., 2012; Ortega et al., 2020; Taebi et al., 2021; Basciano et al., 2011; Kleinstreuer et al., 2012Aramburu et al., 2016; Aramburu et al., 2016; Aramburu et al., 2017; Childress and Kleinstreuer, 2014; Childress and Kleinstreuer, 2014) works investigating them. Aramburu et al. (Aramburu et al., 2017) and Taebi et al. (Taebi et al., 2022) found in their CFD model that the microsphere distribution is especially sensitive to changes in axial location when injecting close to a bifurcation. Injecting from such a location may therefore make branch targeting more difficult and increase the likelihood of a discrepancy between the preliminary scout and therapeutic doses in TARE. This suggests it is best to avoid injecting close to a bifurcation in clinical practice.

Radial injection location is also of great influence on the particle distributions. However, unlike axial injection location this is a difficult parameter to control precisely in clinical practice. Changes in radial position of the catheter during treatment are likely due to the pulsatile flow in the hepatic artery. Unfortunately, no quantitative in vivo or in vitro measurements of catheter position and movement in TARE are currently available. Hence more realistic modeling of the catheter influence in CFD models starts with collection of more experimental data concerning catheter position and movement. For now, the most feasible way to gain more control on the radial injection location, might be the use of an anti-reflux catheter. This catheter type allows radial centering of the catheter tip with respect to the vessel lumen and both in vitro (van den Hoven et al., 2015) and CFD models (Aramburu et al., 2016) suggest that it generates a turbulent flow, leading to a more homogenous downstream microsphere distribution. Even though mixed results were found in vivo (van Roekel et al., 2021; D’Abadie et al., 2021), the possibility to gain control over radial injection location makes it interesting to investigate the possibilities of an anti-reflux catheter in future research. For the same reason, other means of precisely manipulating the catheter position in radioembolization (e.g. actuated ‘steerable’ catheters) could be a compelling topic of future research.

Although the infusion of AT-II seems obsolete (apart from Burton and Gray (1987), Burton et al. (1988), Burton et al. (1989), Burton et al. (1985) no articles were found investigating the influence of AT-II infusion during TARE), it might be a way to influence the microsphere distribution. Several non-TARE related articles were found investigating the vasoconstricting effects of AT-II in the liver vasculature, such as the five human studies included in a systematic review from van den Hoven et al. (2014) (median improvement in T/N blood flow ratio in the range of 1.8 to 3.1). They pointed out that clinical trials are warranted for further investigation of impact of AT-II infusion on treatment efficacy. Therefore, our recommendation would be to further investigate the potentials of AT-II infusion during TARE.

The influence of microsphere shape on the distribution has not been directly investigated. However there has been some research (Aramburu et al., 2017; Smits et al., 2020) concerning the predictive value of Tc-MAA particles used in the pretreatment dose-estimation procedure, which differ in shape from the therapeutic microspheres. These works show mixed results regarding the predictive value of Tc-MAA particles, but it remains unclear whether this is due to the difference in shape. Other factors, such as differences in injection velocity and catheter position between the pretreatment Tc-MAA infusion and the therapeutic microsphere injection, could also be responsible. Dedicated research isolating the effect of microsphere shape is thus needed to evaluate the effect of this parameter.

Microsphere properties such as size and density were found to be of lesser importance (Caine et al., 2017; Amili et al., 2019; Bomberna et al., 2021; Basciano et al., 2010; Ortega et al., 2020; Aramburu et al., 2017). However, all articles investigating this parameter based their conclusions on macroscopic level experiments. Jernigan et al. (Jernigan et al., 2015), is the only article investigating the influence of microsphere properties on a microscopic level and did find a considerable influence, making this a topic for further investigation. Other parameters of low importance were systemic blood flow rate (Caine et al., 2017; Amili et al., 2019; Jernigan et al., 2015; Ortega et al., 2020; Basciano et al., 2011; Childress and Kleinstreuer, 2014), and influence of gravity (Caine et al., 2017; Jernigan et al., 2015). In summary, some of the knowledge gained in the presented studies can be used to improve personalized TARE treatment. In particular, the injection velocity and axial injection location are readily controlled in the clinic and may be tailored to a specific patient. Specific branch targeting of non-approachable distal branches can still be achieved by injecting far from bifurcations with low injection velocities. When multiple downstream branches are to be targeted, the injection velocity can be increased to improve mixing of microspheres with the blood. Other influential parameters such as the radial catheter position first need more accurate methods to measure and control them in the clinic before they can reliably be used to personalize treatment.

### 4.2. Limitations in the models and areas for future research

The complexity of flow and particle dynamics as well as a lack of patient-specific in vivo data makes modeling TARE a difficult task. Simplifying assumptions are warranted to make the problem tractable, but do impose some limitations on the applicability of the results. In the studies included in this systematic review, the most pressing issues relate to the accuracy of boundary conditions specified at the model inlet and outlets.

A first point of attention is the inlet flow rates used in the in vitro studies. These are mostly based on ultrasound measurements. This is not the most reliable technique for determining flow as it assumes a certain velocity profile and the vessel diameter measurements are often inaccurate (Gill, 1985). As a result, we see large deviations in the reported chosen inlet flow rates ranging from 112 ml/min (Jernigan et al., 2015) to 559 ml/min (Richards et al., 2012).

Second, patient specific flow waveforms are often absent due to limited knowledge about the in vivo situation. Only three out of five in vitro studies used pulsatile flow while others assumed a constant inflow. Furthermore, the pulsatile flow waveforms in most studies were based on literature (Basciano et al., 2010) and therefore neglect possible peculiarities in the shape of the waveform for a specific patient. At present it is unclear to what degree such simplifications influence the microsphere distribution. Given the sensitivity of PRMs to local flow features, this is a legitimate concern and crucial information to assess the validity of simulated and experimental results. Therefore, future in vitro and CFD models should investigate the impact of different inlet flow waveforms.

The CFD models employ several simplifications and assumptions to keep computation costs within reasonable bounds. One way to reduce compute time is by simplification and/or truncation of the hepatic arterial geometry. Lertxundi et al. (Lertxundi et al., 2021) and Bomberna et al. (2022) showed that judicious removal of certain parts of the geometry has only a small effect on the simulated microsphere distributions. This supports CFD as a relevant tool in the clinic, where the large computational resources required for detailed simulations are often unavailable.

Another significant reduction in computational cost is achieved by assuming steady blood flow (Kennedy et al., 2010; Bomberna et al., 2021). Childress et al. (Childress and Kleinstreuer, 2014) investigated whether this simplification still led to accurate particle distributions by comparing simulations using pulsatile flow with steady-flow results. Using time-averaged conditions for the steady-flow boundary conditions, the steady-flow results represent the pulsatile flow results with average root mean squared differences (RMSD) of about 17% over the entire cycle. These steady-flow simulations offer a vast savings in compute time, taking only 1% of that for one transient simulation. Steady-flow simulations may therefore be used to obtain quick first insights on the microsphere distributions in a particular geometry but should not be counted on for detailed predictions.

Third, the vast majority of CFD studies assume rigid vessels, as simulating flexible walls is computationally expensive. Childress and Kleinstreuer (2014) performed simulations on a flexible geometry and two rigid geometries and found differences especially during the systolic phase of the cardiac cycle. The authors suggest that the particle injection in the diastolic phase is preferable, as the rigid wall approximation is then more justified. However, this is inconceivable in current clinical practice. Furthermore, the exact way in which the hepatic geometry deforms in vivo is usually unknown, which makes it unlikely that the added computational effort of simulating flexible walls pays off in terms of accuracy of the results.

Finally, the catheter itself is rarely modeled in CFD simulations except in a few cases (Aramburu et al., 2016; Aramburu et al., 2017; Childress et al., 2012; Basciano et al., 2011; Kleinstreuer et al., 2012; Taebi et al., 2021). This simplification may significantly alter the predicted particle trajectories, especially if the injection velocity does not match blood velocity. Catheter-less models may still provide some insight into the flow and particle dynamics, but the results should be interpreted with caution.

In addition to the limitations discussed above, several hurdles must be overcome before PRMs generated by CFD models can be used as a clinical tool for branch targeting. One of the most pressing issues is the sensitivity of the PRMs to the inlet and outlet boundary conditions specified in the model. This is cause for concern as typically only low-quality model input data (e.g. generic values from literature rather than patient-specific measurements) is available. This lack of quality input data may well undermine effective TARE treatment using PRMs. To address this problem, future research should focus on assessing the sensitivity of individual model inputs such as the inlet flow waveform and outlet pressures or resistances. It can then be determined which inputs can be based on generic quantities and for which we need accurate patient-specific measurements.

Finally, we would like to point out the importance of validation of the models. For the CFD models discussed in this review, only two performed in vitro validation (Kennedy et al., 2010; Bomberna et al., 2021). These studies found good agreement between the CFD models and in vitro experiments. However, both works only compared computer models and experiments under steady flow conditions. Additionally, two models (Anton et al., 2021; Roncali et al., 2020) compared with in vivo PET/CT scan data. The results of these in vivo validations were predominantly favorable, supporting the use of CFD models as a means of investigating radioembolization. However, more validation is needed to investigate the models’ applicability in different (patient-specific) scenarios.

### 4.3. Conclusion

The goal of this review was to examine the research on intraprocedural parameters in TARE and identify which parameters strongly impact the microsphere distribution. From the reviewed literature the most important parameters affecting the microsphere distribution are microsphere injection velocity and the radial and axial catheter position. Future research should focus on the parameter sensitivity and the impact on simulated microsphere distributions with respect to the in vitro and CFD model inputs and assumptions. Also, more in vivo and microscopic level data should be collected for validation and to investigate the models’ applicability in patient specific scenarios. In this way, in vitro and CFD models can contribute to a greater understanding of the parameters influencing microsphere distribution in TARE. Ultimately, such models may be used for patient-specific selection of treatment parameters which will improve the efficacy of TARE as treatment for liver cancer.

## Supplementary Material

Supplemental Material
