# Supplementary material for: Transarterial radioembolization: a systematic review on gaining control over the parameters that influence microsphere distribution
Source: Drug Deliv. 2023 Jun 21;30(1):2226366. doi: 10.1080/10717544.2023.2226366 (PMC10286669; doi:10.1080/10717544.2023.2226366)
Supplement: Supplemental Material [file IDRD_A_2226366_SM9152.zip › Supplemental materials_/Supplemental materials_1.docx]

**Supplementary materials**

**Table 1** Detailed search strategy.

| Medline | |
| --- | --- |
| #1 | (SIRT.ti,ab,kf. NOT sirtuin.af.) OR selective internal radiotherapy.ti,ab,kf. OR selective internal radiation therapy.ti,ab,kf. OR selective intra-arterial radiotherapy.ti,ab,kf. OR selective intraarterial radiotherapy.ti,ab,kf. OR selective intra-arterial radiation therapy.ti,ab,kf. OR selective intraarterial radiation therapy.ti,ab,kf. OR radio-embolization.ti,ab,kf. OR radio-embolisation.ti,ab,kf. OR radioembolization.ti,ab,kf. OR radioembolisation.ti,ab,kf. OR TARE.ti,ab,kf. OR trans-arterial radio*.ti,ab,kf. OR transarterial radio*.ti,ab,kf. OR hepatic.mp. OR liver.mp. |
| #2 | ((microsphere*.ti,ab,kf. OR holmium.ti,ab,kf. OR yttrium.ti,ab,kf. OR particle*.ti,ab,kf.) ADJ3 (distribut*.ti,ab,kf. OR dynamic*.ti,ab,kf.))  OR  (Microspheres/ OR exp Holmium/ OR exp Yttrium/ AND (distribut*.ti,ab,kf. OR dynamic*.ti,ab,kf.)) |
| #3 | 1 AND 2 |
| #4 | 3 NOT (nanoparticle* or nano-particle* or nano particle* or nanosphere* or nano-sphere* or nano sphere*).ti,ab,kf. |
| Embase | |
| #1 | radioembolization/  OR  ((SIRT.ti,ab,kw. NOT sirtuin.af.) OR selective internal radiotherapy.ti,ab,kw. OR selective internal radiation therapy.ti,ab,kw. OR selective intra-arterial radiotherapy.ti,ab,kw. OR selective intraarterial radiotherapy.ti,ab,kw. OR selective intra-arterial radiation therapy.ti,ab,kw. OR selective intraarterial radiation therapy.ti,ab,kw. OR radio-embolization.ti,ab,kw. OR radio-embolisation.ti,ab,kw. OR radioembolization.ti,ab,kw. OR radioembolisation.ti,ab,kw. OR TARE.ti,ab,kw. OR trans-arterial radio*.ti,ab,kw. OR transarterial radio*.ti,ab,kw. OR hepatic.mp. OR liver.mp.) |
| #2 | ((microsphere*.ti,ab,kw. OR holmium.ti,ab,kw. OR yttrium.ti,ab,kw. OR particle*.ti,ab,kw.) ADJ3 (distribut*.ti,ab,kw. OR dynamic*.ti,ab,kw.))  OR  (Microspheres/ OR exp Holmium/ OR exp Yttrium/ AND (distribut*.ti,ab,kw. OR dynamic*.ti,ab,kw.)) |
| #3 | 1 AND 2 |
| #4 | 3 NOT (nanoparticle* or nano-particle* or nano particle* or nanosphere* or nano-sphere* or nano sphere*).ti,ab,kw. |
| #5 | Limit 4 to conference abstract status |
| #6 | 4 NOT 5 |
| Web of Science | |
| #1 | TS=(("SIRT" NOT "sirtuin") OR "selective internal radiotherapy" OR "selective internal radiation therapy" OR "selective intraarterial radiotherapy" OR "selective intra-arterial radiation therapy" OR "selective intraarterial radiation therapy" OR "radio-embolization" OR "radio-embolisation" OR "radioembolization" OR "radioembolisation" OR "TARE" OR "trans-arterial radio*" OR "transarterial radio*" OR "hepatic" OR "liver") |
| #2 | TS=(("microsphere*" OR "holmium" OR "yttrium" OR "particle") NEAR/2 ("distribut*" OR "dynamic*")) |
| #3 | #1 AND #2 |
| #4 | # 3 NOT TS=("nanoparticle*" OR "nano-particle*" OR "nano particle*" OR "nanosphere*" OR "nano-sphere*" OR "nano sphere*") |
