# Supplementary material for: Transarterial radioembolization: a systematic review on gaining control over the parameters that influence microsphere distribution
Source: Drug Deliv. 2023 Jun 21;30(1):2226366. doi: 10.1080/10717544.2023.2226366 (PMC10286669; doi:10.1080/10717544.2023.2226366)
Supplement: Supplemental Material [file IDRD_A_2226366_SM9152.zip › Supplemental materials_/Supplemental materials_2.docx]

**Supplementary materials**

Table 2 and 3 provide overviews of the investigated parameters and most important outcomes of respectively the in vitro and CFD studies.

**Table 2** In vitro: overview of the investigated parameters and most important outcomes.

| **In vitro model** | **Model description** | **Investigated parameters** | **Reported outcomes** |
| --- | --- | --- | --- |
| Richards 2012 [1] | Flat model, a parent vessel bifurcates into five outlets. Diameters based on literature [2-4]. | 26 experiments were performed at several locations within the injection plane and compared to computer simulations. | 1) The experimental microsphere distribution was highly dependent on the radial injection location, and thus individual branch-targeting was possible.  2) 14 of 26 injection sites (54%) correlated with the computer simulations. After a vertical offset of -1.57 mm and scaling of 15%, 23 of 26 distributions matched (88%), which illustrates the similarity between the experiments and simulations. |
| Jernigan 2015 [5] | Flat model, a parent vessel bifurcates into sixteen outlets. One outlet connected to a planar tumour model to study penetration depth of microspheres on microlevel (up to 15 µm). Diameters based on 3D CT imaging and anatomic measurements of vessel diameters. | Microsphere type (resin vs. glass), systemic flow rate, microsphere injection velocity, and inclination angle on penetration distance | 1) Penetration depth of resin microspheres was significantly higher compared to glass microspheres (45.1 cm $\pm$ 11.8 vs 22.3 cm $\pm$ 9.9, P = .005).  2) For all systemic flow rates (112, 160, 200 mL/min) similar results were observe (P = .9583 for glass, P = .2249 for resin).  3) For glass microspheres: average penetration depth was much higher for the maximum injection velocity (36 mL/min) compared to the minimum injection velocity (18 mL/min), though not statistically significant (29.5 cm $\pm$ 9.1 vs 22.6 cm $\pm$ 10.3).  4) Inclination angle (-22.5$^{\circ}$ downward flow up to 22.5$^{\circ}$ upward flow) had no statistically significant effect (P = .8013 for glass, P = .1261 for resin). |
| Caine 2017 [6] | Flat model, a parent vessel bifurcates into six outlets. Diameters based on angiographic images and vessel diameters from literature. | Microsphere type (resin vs. glass), model orientation (vertical vs. horizontal), BMF flow rate, microsphere injection velocity, injection solution viscosity | 1) Substantially equivalent distribution in vertical and horizontal orientations of the 3D model were found for glass (20.05% vs 24.19%, P = .0999) and resin (17.21% vs 21.25%, P = .0622) microspheres. However, total flow volume did distribute differently in the vertical orientation; more flow was going to the posterior vessels (18.83% vs 13.05%, P < .05).  2) Gravity had no influence on the resin vs. glass microsphere distribution in the vertical orientation of the 3D model.  3) Correlation between microsphere distribution and injection fluid distribution: moderate (r^2^ = 0.5712; n = 144), correlation between microsphere distribution and total fluid volume: poor (r^2^ = 0.0104; n = 144).  4) Reducing injection velocity and BMF flow rate showed equivalent microsphere distribution for glass and resin (19.81% vs 24.26%, P =.182), which highlights the minimal influence of microsphere density.  5) In case of saline solution as injection solution, microsphere injection velocities > 10 ml/min were found to enhance mixing between the microspheres and the total fluid volume. Increasing the viscosity of the injection solution required higher injection flow rates to achieve satisfactory mixing.  6) Injection solutions can be modified (increasing the viscosity) with up to 30% vol/vol contrast agent for angiographic visualization. Above this level, significant loss of microsphere alignment with the total fluid volume was observed. |
| Van den Hoven 2015 [7] | Model described by Jernigan et al. An extra piece of surgical tubing was inserted at the catheter tip position to optimize vessel sealing by the anti-reflux catheter. | Differences between a standard microcatheter and an anti-reflux catheter, expressed in flow pattern and downstream branch distribution. | 1) The standard microcatheter showed an ordered outflow pattern, consistent with laminar flow, while the anti-reflux catheter had a chaotic outflow pattern, consistent with turbulent flow.  2) A significantly more homogenous distribution of microspheres was found with the anti-reflux catheter compared to the standard microcatheter. The median deviation from homogeneous distribution (DHD; distance to a perfect homogeneous distribution (50%-50%) in percentage points was 15.54% for the anti-reflux catheter and 40.85% for the standard microcatheter.  3) For the standard microcatheter, it seemed that the homogeneity of the downstream distribution was dependent on the radial catheter position. In the experiments with a centred radial catheter position, a relatively homogeneous distribution was found (DHD 8.13-10.50%), while for the experiments with an off-centred radial catheter position the distribution was more skewed (DHD 29.68-50%). |
| Amili 2019 [8] | Flat model, a parent vessel bifurcates symmetrically four times into sixteen outlets. Diameters based on literature [2-4]. | Systolic vs. cycle-averaged flow rates, particle diameter, uniform outflow vs. double outflow in 2 adjacent outlets. | 1) Regardless of the chosen flow rate, the particle distribution among the outlets is fundamentally different (ranging 0.005 – 0.12) from what would be expected based on the symmetrical flow rate distribution (each outlet 1/16 = 0.0625).  2) Smaller (less inertial) particles do not necessarily better follow the flow compared to larger particles; this study found that distribution of smaller particles can deviate more from volumetric flow rate compared to larger particles and it was linked to their radial release location along the main vessel.  3) Branches receiving double flow rate, showed an increase in the number of particles. However, this was mostly observed when two of the outer outlets received doubled flow. In case the inner outlets received more flow rate, this effect differed. |
| Bomberna 2020 [9] | 3D geometry of a human cirrhotic arterial tree obtained via vascular corrosion casting followed by micro-CT scanning. | Comparison with computer simulations in two experiments. Experiment 1: microspheres were mixed with water in the main reservoir, and pumped into the hepatic artery model.  Experiment 2: manual injection of microspheres close to the inlet. | 1) Average difference per reservoir between experimental particle distribution and fluid distribution was 2.45% and 2.14% for experiment 1 and 2, respectively.  2) On average for a collecting reservoir, the difference between experiments and computer simulations was 2.73% and 2.97% for experiment 1 and 2, respectively. |
| Miller 2022 [10] | Flat model, a parent vessel bifurcates into sixteen outlets. One outlet connected to a planar tumour model with four identical microvascular trees (18 µm wide outlet arterioles). | Comparison of two delivery devices: a dual-syringe (DS) system (delivered a relatively constant microsphere concentration throughout administration) and a bolus delivery (BD) system (equivalent to clinical administration method). | 1) Microsphere concentration: BD system showed high microsphere concentration peaks with quick drop in concentrations, while DS system showed a slower rise to peak with a longer steady plateau.  2) Microsphere distribution: BD system showed significant proximal accumulation of microspheres, while DS system showed more distal filling of the vasculature. |

***Table 3*** *CFD: overview of the investigated parameters and most important outcomes*

| **CFD model** | **Model description** | **Investigated parameters** | **Reported Outcomes** |
| --- | --- | --- | --- |
| Kennedy 2010 [11] | Steady flow in a representative hepatic artery system consisting of a parent vessel that bifurcates into five outlets. | Outlet pressures  Uniform and parabolic particle-injection distributions at the inlet plane | 1) Decreased outlet pressure leads to substantially larger blood flow rates to the corresponding outlets, corresponding to the case of increased blood flow to tumour vessels.  2) Particle injection distribution has a large influence on the fraction of particles exiting each outlet.  Blood flow rates and particle flow rates to each outlet do not necessarily match. |
| Basciano 2010 [12] | Pulsatile flow in a representative hepatic artery system consisting of a parent vessel that bifurcates into five outlets. | Particle injection timing  Restitution coefficient of particle-wall collisions  Radial release position as it varies with time (transient particle release map)  Microsphere properties | 1) When injecting particles during the decelerating phase of the pulsating flow, particle properties have more influence on the distribution between outlets.  2) The restitution coefficient (indicating the degree of damping in particle-wall collisions) had little effect on particle distributions  3) Using “transient backtracking”, time-dependent particle release maps can be generated and used for targeting specific vessels. |
| Basciano 2011 [13] | Pulsatile flow in representative and patient-inspired hepatic arterial systems. The representative arterial system consists of a parent vessel that bifurcates into five outlets. The patient-inspired system is a tortuous geometry consisting of an inlet and three outlets. | Downstream resistances at the outlets  Flow waveform  Particle injection timing  Injection velocity  Influence of catheter | 1) Downstream resistances strongly influence the flow distribution.  2) Although flow waveform leads to notable differences in the velocity field, their influence is of lesser importance than for example arterial geometry  3) Injection timing with respect to the cardiac cycle has a strong influence on particle distribution.  4) For large injection velocities, particles cross flow streamlines.  Catheter presence alters the particle release maps. |
| Kleinstreuer 2012 [14] | Pulsatile flow in a representative hepatic artery system consisting of a parent vessel that bifurcates into five outlets. | Influence of a smart micro catheter with support struts on flow and particles transport  Microsphere injection velocity | 1) The catheter and support struts lead to a local disturbance of the flow field. This subsequently leads to changes in the particle trajectories.  2) Further downstream from the catheter the flow disturbance damps out.  3) Very low microsphere injection velocities can lead to microspheres being trapped in flow recirculation regions close to the catheter tip.  In contrast, injection velocities much higher than the local flow velocity enable particles to cross flow streamlines. This makes microsphere trajectories unpredictable as inertial effects dominate. |
| Childress 2012 [15] | Pulsatile flow in a patient-inspired hepatic artery system consisting of a parent vessel that bifurcates into five outlets. | Radial catheter positions  Effect of catheter  Particle release timing (temporal release intervals)  Injection profile (step, ramp, S-curve) | 1) Catheter presence leads to local disturbances in flow field which affect particle trajectories  2) Particle release maps varied considerably throughout the pulse, making it difficult to target a particular branch if particles are injected throughout the pulse.  The step injection profile leads to particles spreading out from the initial release location. This could make it more difficult to target a particular branch if the corresponding zone in the particle release map is small. |
| Childress 2014 [16] | Pulsatile and steady flow in a representative hepatic artery system consisting of a parent vessel that bifurcates into five outlets. | Extent to which pulsatile flow and particle simulations can be approximated using steady flow. | 1) Due to small variations in flow rate during diastolic phase of the pulsatile flow, injection during this phase can be reasonably approximated using steady conditions.  2) Time-averaged data can be used to generate the steady conditions used to model pulsatile flow. In complex cases, simulating multiple representative steady flow scenarios is still an efficient way to determine a suitable injection location.  Injection should be avoided during intervals of high acceleration or deceleration as the particle release maps fluctuate rapidly, making accurate targeting difficult. |
| Childress 2014 [17] | Pulsatile flow in a representative hepatic artery system consisting of a parent vessel that bifurcates into five outlets. | Impact of flexible walls (fluid-structure interaction) on blood flow and microspheres transport | Wall flexibility leads to lower flow rates during systole yet higher flowrates during diastole due to the Windkessel effect.  The flexible geometry can be reasonably approximated by a rigid geometry in the diastolic phase. The rigid geometry in this case can be taken to be the time-averaged geometry over the entire diastolic phase. |
| Aramburu 2015 [18] | Pulsatile flow in a patient-specific hepatic artery consisting of one inlet and 29 outlets. Circular vessel cross sections were constructed. | Determining physiological outflow boundary conditions based on known perfusion rates of healthy and tumorous tissue | Paper presents a method to derive inlet and outlet boundary conditions based on tumour and healthy liver perfusion rates. Steady-state simulations and 0D modelling can then be used to derive a matching physiological pressure field. |
| Aramburu 2016 [19] | Pulsatile flow in a patient-specific hepatic artery consisting of one inlet and 29 outlets. Circular vessel cross sections were constructed. | Effect of catheter type: anti-reflux vs standard end-hole catheter.  Axial catheter tip location. | 1) Injecting via a standard catheter leads to particles smoothly entering the bloodstream whereas the anti-reflux catheter leads to a more rapid spreading of the microspheres over the cross section.  2) At the inlet position the catheter type led to a difference in particle distributions. However close to a bifurcation there was little difference. |
| Aramburu 2016 [20] | Pulsatile flow in a patient-specific hepatic artery consisting of one inlet and 29 outlets. Circular vessel cross sections were constructed. | Effect of microcatheter distal direction.  Microsphere injection velocity  Injection point within cross section | 1) Injection point within the cross section has a large influence on the particle distributions  2) The distal microsphere direction has an influence on the flow near the catheter. This led to notable differences in particle distributions in some configurations  The ratio of microsphere injection velocity to blood flow velocity determines whether particles will cross flow streamlines. Especially when injecting during the diastolic phase the ratio may become large enough to affect particle trajectories. |
| Aramburu 2017 [21] | Simulation of radioembolization pre-treatment and actual treatment under pulsatile flow in a patient-specific geometry consisting of one inlet and 29 outlets. Circular vessel cross sections were constructed. | Particle properties (Tc-MAA and Y90 resin microspheres)  Axial catheter tip position  Cancer burden (blood flow rates) | 1) The differences in properties of the Tc-MAA and Y90 particles only led to small differences in distributions. This supports the use of Tc-MAA in the pre-treatment procedure.  2) A small shift in catheter position leads to vast differences in particle distributions, especially when injecting near a bifurcation.  Increased cancer burden usually leads to a greater number of microspheres arriving at the tumour. However, when particle injection velocities are large compared to systemic velocity, particle inertia effects may lead the particle distributions to differ from flow distribution. |
| Simoncini 2017 [22] | Segmentation of patient hepatic vasculature and simulation of blood flow. Validation with phase contrast MRI. Patient-specific geometries from four patients were constructed using cone beam CT 3D angiography. The geometries have between 20 and 50 outlets. | Validation of segmentation method and choice of boundary conditions | Application of the segmentation method using cone-beam CT angiography data and outlet boundary conditions proportional to vessel diameters leads to results coherent with literature. |
| Aramburu 2017 [23] | Pulsatile flow in a patient-specific hepatic artery consisting of one inlet and 29 outlets with explicit modelling of an angled-tip micro catheter. Circular vessel cross sections were constructed. | Tip orientation of the angled-tip microcatheter  Microsphere injection velocity | 1) Orientation of the catheter tip leads to changes in local flow dynamics which may affect microsphere distributions.  2) Because microsphere trajectories depend heavily on the intricacies of the flow field, orienting the tip towards a certain branch does not necessarily increase microsphere distribution to this branch.  3) Increased injection velocity leads to faster particle mixing in the blood flow. |
| Roncali 2020 [24] | Two patient-specific geometry from cone beam CT data combined with fluid flow simulation and a dosimetry model. The two geometries had 23 and 46 outlets. Microspheres are assumed to follow flow streamlines. | Proof of concept of integrated CFDose workflow using PET imaging for validation. | 1) The simulated dose distribution was within 10% of that derived from PET imaging.  2) Realistic results for the blood flow distribution require patient-specific computations. Use of Poiseuille’s law (Simoncini et al. [22]) will lead to less accurate predictions of the dose distribution. |
| Ortega 2020 [25] | Pulsating flow and particles transport in an idealized symmetrical geometry modelled after the most common configuration of the hepatic artery. The domain consists of three bifurcations leading to 8 outlets. | Use of idealized geometry as a valid research tool under different kinds of boundary conditions  Injection velocity  Microsphere properties / type of microspheres | 1) Physiologically realistic inflow boundary conditions are needed in idealized models for them to provide a valid research tool.  2) Larger injection velocities lead to more rapid mixing of microspheres with the flow.  3) Microsphere properties have only a small effect on microsphere distribution. |
| Taebi 2020 [26] | Pulsating flow and microsphere transport in a patient-specific geometry based on cone beam CT with 46 outlets. | Effect of outlet resistance boundary conditions | 1) Changing the total resistance of outlet BC’s has a significant effect on the flow field and microsphere trajectories and distribution.  The ratio of distal to proximal resistances in the 3-element Windkessel model used had a much smaller impact on flow and microsphere transport. |
| Taebi 2021 [27] | Pulsating flow and in a patient-specific geometry based on cone beam CT (46 outlets), including a dosimetry model to predict radiation dose. Microspheres distribution is assumed to follow flow distribution. | Injection location (comparison of location in right hepatic artery and further downstream) | 1) The injection location further downstream led to 82% of flow to the tumour outlets. The injection in the right hepatic artery led to 22% of flow to these outlets.  The nonhomogeneous distribution of microspheres between liver segments shows the importance of patient-specific dosimetry methods. |
| Bomberna 2021 [9] | Steady flow in two patient-specific liver geometries: one healthy (16 outlets) and one cirrhotic (21 outlets). The geometries were obtained using vascular corrosion casting followed by micro-CT scanning of the casts. | Axial and in-plane injection location  Microsphere properties  Cancer burden (healthy and cirrhotic liver) | 1) Axial injection location has a significant impact on particle distribution.  2) Choice of in-plane injection location can be used to target specific branches.  3) Particle size and density have limited potential to alter the microsphere distribution.  4) Cancer burden generally leads to increased flow and microspheres towards the tumours. However, the size of the effect will depend on the patient-specific geometry. |
| Lertxundi 2021 [28] | Simulation of pulsatile flow and microsphere transport in three patient specific hepatic geometries, each under with varying degrees of truncation. | Effect of truncating the geometry to reduce computational cost of the model | With the developed rule for truncation of geometry, microsphere distributions accurate to within 10% of the baseline model were obtained with an average reduction in computational time of 62%. |
| Anton 2021 [29] | Simulation of pulsatile flow and microspheres transport in three patient specific hepatic geometries with validation of predicted dose using PET/CT imaging. | Proof of concept of the ability for personalized CFD models to accurately predict microsphere distribution. | Good agreement between the actual microsphere distribution derived from PET/CT scans and the simulated distribution was obtained. Average differences between measured and simulated distributions were 2.36%, 2.02% and 4.12%. The largest discrepancy was obtained for a geometry in which the injection point was close to a bifurcation which is the suspected reason for the reduced accuracy of results. |
| Taebi 2021 [30] | Modelling of catheter presence via boundary conditions in the trunk of a patient-specific right hepatic arterial tree | Catheter presence (modelled via inlet boundary condition) | The presence of a catheter affects the local flow field, leading to a deviation from the parabolic profile observed in absence of a catheter. |
| Taebi 2022 [31] | Modelling of transient flow in a patient-specific geometry and assuming microspheres follow flow streamlines. | Sensitivity of microsphere distribution to distance from catheter tip to bifurcations. | When injecting close to the bifurcation, changes in catheter position have a (very) large effect on microsphere distributions. The sensitivity becomes smaller when injecting farther from a bifurcation. In this study, changes in microsphere distribution of each outlet were less than 10% for three radial injection positions at an axial position located three times the right hepatic artery diameter proximal to the first bifurcation. |
| Ortega 2022 [32] | Modelling of flow and particles transport in a generalized 3D liver geometry. The catheter is explicitly modelled. | Effect of several novel designs of side-hole catheters on microsphere distributions and comparison to standard end-hole catheter. | Side-hole catheters with a closed frontal tip lead to more rapid dispersion of particles across the vessel lumen. To achieve this effect it is important to use an injection velocity sufficiently large for particles to cross flow streamlines. The more rapid mixing leads to microsphere distributions which more closely follow the flow distribution. |
| Bomberna 2022 [33] | Hybrid particle-flow model which only models flow and particle trajectories in the first few bifurcations and assumes particles follow blood flow split in the smaller, more distal vessels. | Evaluation of an approach to reduce computational effort by truncating the geometry and assuming particles follow the flow distribution in the truncated vessels. This “hybrid” particle-flow model is compared to flow and particle simulations in the un-truncated geometry. | The particle distributions using the hybrid particle-flow model closely approximate those using calculated particle-flow simulations in the full geometry. Median differences between the full and hybrid model are less than 0.30% in the geometry investigated here, with a maximum outlet-specific difference of 3.50%. |

**Bibliography**

1. Richards AL, Kleinstreuer C, Kennedy AS, Childress E, Buckner GD. Experimental microsphere targeting in a representative hepatic artery system. IEEE Trans Biomed Eng. 2012;59:198-204. doi:<https://dx.doi.org/10.1109/TBME.2011.2170195>.

2. Ishigami K, Zhang Y, Rayhill S, Katz D, Stolpen A. Does variant hepatic artery anatomy in a liver transplant recipient increase the risk of hepatic artery complications after transplantation? AJR Am J Roentgenol. 2004;183:1577-84. doi:10.2214/ajr.183.6.01831577.

3. Carlisle KM, Halliwell M, Read AE, Wells PN. Estimation of total hepatic blood flow by duplex ultrasound. Gut. 1992;33:92-7. doi:10.1136/gut.33.1.92.

4. Han SH, Rice S, Cohen SM, Reynolds TB, Fong TL. Duplex Doppler ultrasound of the hepatic artery in patients with acute alcoholic hepatitis. J Clin Gastroenterol. 2002;34:573-7. doi:10.1097/00004836-200205000-00019.

5. Jernigan SR, Osborne JA, Mirek CJ, Buckner G. Selective internal radiation therapy: quantifying distal penetration and distribution of resin and glass microspheres in a surrogate arterial model. J Vasc Interv Radiol. 2015;26:897-904.e2. doi:<https://dx.doi.org/10.1016/j.jvir.2015.02.022>.

6. Caine M, McCafferty MS, McGhee S, Garcia P, Mullett WM, Zhang X, et al. Impact of Yttrium-90 Microsphere Density, Flow Dynamics, and Administration Technique on Spatial Distribution: Analysis Using an In Vitro Model. J Vasc Interv Radiol. 2017;28:260-8.e2. doi:<https://dx.doi.org/10.1016/j.jvir.2016.07.001>.

7. van den Hoven AF, Lam MG, Jernigan S, van den Bosch MA, Buckner GD. Innovation in catheter design for intra-arterial liver cancer treatments results in favorable particle-fluid dynamics. J Exp Clin Cancer Res. 2015;34:74. doi:<https://dx.doi.org/10.1186/s13046-015-0188-8>.

8. Amili O, Golzarian J, Coletti F. In Vitro Study of Particle Transport in Successively Bifurcating Vessels. Annals of Biomedical Engineering. 2019;47:2271-83. doi:<https://dx.doi.org/10.1007/s10439-019-02293-2>.

9. Bomberna T, Koudehi GA, Claerebout C, Verslype C, Maleux G, Debbaut C. Transarterial drug delivery for liver cancer: numerical simulations and experimental validation of particle distribution in patient-specific livers. Expert Opin Drug Deliv. 2021;18:409-22. doi:<https://dx.doi.org/10.1080/17425247.2021.1853702>.

10. Miller SR, Jernigan SR, Abraham RJ, Buckner GD. Comparison of Bolus and Dual Syringe Administration on Glass Yttrium-90 Microsphere Deposition in an In Vitro Microvascular Hepatic Tumor Model. J Vasc Interv Radiol. 2022. doi:10.1016/j.jvir.2022.07.032.

11. Kennedy AS, Kleinstreuer C, Basciano CA, Dezarn WA. Computer modeling of yttrium-90-microsphere transport in the hepatic arterial tree to improve clinical outcomes. Int J Radiat Oncol Biol Phys. 2010;76:631-7. doi:<https://dx.doi.org/10.1016/j.ijrobp.2009.06.069>.

12. Basciano CA, Kleinstreuer C, Kennedy AS, Dezarn WA, Childress E. Computer Modeling of Controlled Microsphere Release and Targeting in a Representative Hepatic Artery System. Annals of Biomedical Engineering. 2010;38:1862-79. doi:10.1007/s10439-010-9955-z.

13. Basciano C, Kleinstreuer C, Kennedy A. Computational Fluid Dynamics Modeling of 90Y Microspheres in Human Hepatic Tumors. Journal of Nuclear Medicine & Radiation Therapy. 2011;01. doi:10.4172/2155-9619.1000112.

14. Kleinstreuer C, Basciano CA, Childress EM, Kennedy AS. A new catheter for tumor targeting with radioactive microspheres in representative hepatic artery systems. Part I: impact of catheter presence on local blood flow and microsphere delivery. J Biomech Eng. 2012;134:051004. doi:10.1115/1.4006684.

15. Childress EM, Kleinstreuer C, Kennedy AS. A new catheter for tumor-targeting with radioactive microspheres in representative hepatic artery systems--part II: solid tumor-targeting in a patient-inspired hepatic artery system. J Biomech Eng. 2012;134:051005. doi:<https://dx.doi.org/10.1115/1.4006685>.

16. Childress EM, Kleinstreuer C. Computationally efficient particle release map determination for direct tumor-targeting in a representative hepatic artery system. J Biomech Eng. 2014;136:011012.

17. Childress EM, Kleinstreuer C. Impact of fluid-structure interaction on direct tumor-targeting in a representative hepatic artery system. Annals of Biomedical Engineering. 2014;42:461-74. doi:<http://dx.doi.org/10.1007/s10439-013-0910-7>.

18. Aramburu J, Antón R, Bernal N, Rivas A, Ramos JC, Sangro B, et al. Physiological outflow boundary conditions methodology for small arteries with multiple outlets: a patient-specific hepatic artery haemodynamics case study. Proc Inst Mech Eng H. 2015;229:291-306. doi:10.1177/0954411915578549.

19. Aramburu J, Anton R, Rivas A, Ramos JC, Sangro B, Bilbao JI. Computational assessment of the effects of the catheter type on particle-hemodynamics during liver radioembolization. J Biomech. 2016;49:3705-13. doi:<https://dx.doi.org/10.1016/j.jbiomech.2016.09.035>.

20. Aramburu J, Antón R, Rivas A, Ramos J, Sangro B, Bilbao J. Numerical investigation of liver radioembolization via computational particle–hemodynamics: The role of the microcatheter distal direction and microsphere injection point and velocity. J Biomech. 2016;49. doi:10.1016/j.jbiomech.2016.09.034.

21. Aramburu J, Anton R, Rivas A, Ramos JC, Sangro B, Bilbao JI. Computational particle-haemodynamics analysis of liver radioembolization pretreatment as an actual treatment surrogate. International Journal for Numerical Methods in Biomedical Engineering. 2017;33:02. doi:<https://dx.doi.org/10.1002/cnm.2791>.

22. Simoncini C, Rolland Y, Morgenthaler V, Jurczuk K, Saint-Jalmes H, Eliat PA, et al. Blood Flow Simulation in Patient-Specific Segmented Hepatic Arterial Tree. Irbm. 2017;38:120-6. doi:<http://dx.doi.org/10.1016/j.irbm.2017.04.001>.

23. Aramburu J, Anton R, Rivas A, Ramos JC, Sangro B, Bilbao JI. The role of angled-tip microcatheter and microsphere injection velocity in liver radioembolization: A computational particle-hemodynamics study. International Journal for Numerical Methods in Biomedical Engineering. 2017;33:12. doi:<https://dx.doi.org/10.1002/cnm.2895>.

24. Roncali E, Taebi A, Foster C, Vu CT. Personalized Dosimetry for Liver Cancer Y-90 Radioembolization Using Computational Fluid Dynamics and Monte Carlo Simulation. Annals of Biomedical Engineering. 2020;48:1499-510. doi:<https://dx.doi.org/10.1007/s10439-020-02469-1>.

25. Ortega J, Anton R, Ramos JC, Rivas A, Larraona GS, Sangro B, et al. On the importance of spiral-flow inflow boundary conditions when using idealized artery geometries in the analysis of liver radioembolization: A parametric study. International Journal for Numerical Methods in Biomedical Engineering. 2020;36:e3337. doi:<https://dx.doi.org/10.1002/cnm.3337>.

26. Taebi A, Pillai RM, Roudsari BS, Vu CT, Roncali E. Computational Modeling of the Liver Arterial Blood Flow for Microsphere Therapy: Effect of Boundary Conditions. Bioengineering (Basel). 2020;7:29. doi:<https://dx.doi.org/10.3390/bioengineering7030064>.

27. Taebi A, Vu CT, Roncali E. Multiscale Computational Fluid Dynamics Modeling for Personalized Liver Cancer Radioembolization Dosimetry. J Biomech Eng. 2021;143:01. doi:<https://dx.doi.org/10.1115/1.4047656>.

28. Lertxundi U, Aramburu J, Ortega J, Rodriguez-Fraile M, Sangro B, Bilbao JI, et al. CFD Simulations of Radioembolization: A Proof-of-Concept Study on the Impact of the Hepatic Artery Tree Truncation. Mathematics. 2021;9:21. doi:10.3390/math9080839.

29. Anton R, Antonana J, Aramburu J, Ezponda A, Prieto E, Andonegui A, et al. A proof-of-concept study of the in-vivo validation of a computational fluid dynamics model of personalized radioembolization. Scientific Reports. 2021;11:3895. doi:<https://dx.doi.org/10.1038/s41598-021-83414-7>.

30. Taebi A, Berk S, Roncali E. Realistic boundary conditions in SimVascular through inlet catheter modeling. BMC Res Notes. 2021;14:215. doi:10.1186/s13104-021-05631-7.

31. Taebi A, Janibek N, Goldman R, Pillai R, Vu CT, Roncali E. The Impact of Injection Distance to Bifurcations on Yttrium-90 Distribution in Liver Cancer Radioembolization. Journal of Vascular and Interventional Radiology. 2022;33:668-77.e1. doi:<https://doi.org/10.1016/j.jvir.2022.03.006>.

32. Ortega J, Antón R, Ramos JC, Rivas A, S. Larraona G, Sangro B, et al. Computational study of a novel catheter for liver radioembolization. International Journal for Numerical Methods in Biomedical Engineering. 2022;38:e3577. doi:<https://doi.org/10.1002/cnm.3577>.

33. Bomberna T, Vermijs S, Lejoly M, Verslype C, Bonne L, Maleux G, et al. A Hybrid Particle-Flow CFD Modeling Approach in Truncated Hepatic Arterial Trees for Liver Radioembolization: A Patient-specific Case Study. Front Bioeng Biotechnol. 2022;10:914979. doi:10.3389/fbioe.2022.914979.
